# Broad Neutralizing Activity of Monoclonal Antibodies Against the Omicron Variants Isolated From Patients With Early Severe Acute Respiratory Syndrome Coronavirus‐2

**DOI:** 10.1002/jmv.70969

**Published:** 2026-05-13

**Authors:** Da Sol Kim, Uijin Kim, Hye‐Min Woo, Hansaem Lee, Eun‐Seong Jo, Min Jeong Noh, So‐Young Lee, Byoung Kwon Park, Jeong‐Sun Yang, Kyung‐Chang Kim, Joo‐Yeon Lee, Dong‐Min Wang, Hyun‐Soo Cho, Hyun‐Joo Kim

**Affiliations:** ^1^ Division of Emerging Virus and Vector Research, Center for Emerging Virus Research, Korea National Institute of Health Korea Disease Control and Prevention Agency Cheongju‐si Republic of Korea; ^2^ Department of Systems Biology, Division of Life Sciences Yonsei University Seoul Republic of Korea; ^3^ Center for Emerging Virus Research, Korea National Institute of Health Korea Disease Control and Prevention Agency Cheongju‐si Republic of Korea

**Keywords:** combination, cryo‐EM, monoclonal antibodies, neutralizing, SARS‐CoV‐2

## Abstract

Since its emergence in 2019, severe acute respiratory syndrome coronavirus‐2 (SARS‐CoV‐2) has caused a global pandemic driven by rapid mutation and high transmissibility. Current therapeutic strategies may not effectively address the continuously evolving mutant strains, underscoring the need for treatments with broad neutralizing activity. Two monoclonal antibodies (mAbs): SR‐23 and CS‐42, isolated from convalescent patients infected with SARS‐CoV‐2 during the early phase of the 2020 pandemic, demonstrated significant neutralizing activities against a variety of variants. Specifically, SR‐23 exhibited neutralizing activity against BA.5 and XBB1.5, whereas CS‐42 showed efficacy against Delta, BA.1, and BA.2 variants. In addition, combining the two mAbs demonstrated improved neutralization activity. Cryo‐electron microscopy (cryo‐EM) structural analysis revealed that SR‐23 and CS‐42 bind to nonoverlapping epitopes on the SARS‐CoV‐2 spike protein. Furthermore, both antibodies exhibited strong therapeutic effects in K18‐hACE2 mice infected with D614G, BA.2, and XBB.1.5. These findings provide insights into the development of antibody‐based therapeutics targeting emerging Omicron variants and suggest that combined administration could broaden neutralizing activity.

## 1 Introduction

Severe acute respiratory syndrome coronavirus‐2 (SARS‐CoV‐2) emerged in 2019 and rapidly spread worldwide, causing the coronavirus disease‐2019 (COVID‐19) pandemic [1]. Since then, multiple variants have been reported globally [2], resulting in over 779 million confirmed cases and more than 7.1 million deaths [3].

The SARS‐CoV‐2 spike protein contains a furin cleavage site between the S1 and S2 subunits. The S1 subunit, comprising the receptor‐binding domain (RBD) and N‐terminal domain, binds to the angiotensin‐converting enzyme 2 (ACE2) receptor, while the S2 subunit mediates membrane fusion for viral entry [4, 5]. Given its crucial role in viral entry, the spike protein is the primary target of both antibody‐based therapeutics and vaccines, as well as the natural neutralizing antibody response induced infection [6, 7].

Monoclonal antibody (mAb) therapies, including casirivimab, imdevimab, bamlanivimab, etesevimab, sotrovimab, and regdanvimab, inhibit viral entry by blocking the interaction between the spike protein and ACE2, while SP1‐77 suppresses viral entry by preventing membrane fusion [8, 9, 10, 11]. However, viral mutations have reduced the neutralizing efficacy of antibodies developed early in the pandemic [12, 13]. To overcome this, combination antibody therapies, such as REGEN‐COV (casirivimab + imdevimab), were designed for broader neutralization [14].

The aim of this study was to identify mAbs derived from early‐convalescent COVID‐19 patients and to assess their neutralizing activity against various SARS‐CoV‐2 variants. Broadly neutralizing antibodies naturally produced during early infection may offer valuable templates for developing next‐generation therapeutics and improving preparedness against future viral threats.

## 2 Materials and Methods

### 2.1 Clinical Specimen Collection

Blood samples were obtained from six recovered COVID‐19 patients in Korea between February and April 2020 with approval from the Institutional Review Board of Seoul National University Hospital (IRB No. 2002‐105‐110). Peripheral blood mononuclear cells (PBMCs) and serum were separated from the collected blood samples using Ficoll‐Paque (GE Healthcare, Waukesha, USA) according to the manufacturer's instructions.

### 2.2 Cell Lines

Vero E6 cells were cultured in Dulbecco's modified Eagle's medium (DMEM) supplemented with 10% heat‐inactivated fetal bovine serum (FBS) and 1% penicillin/streptomycin (P/S). 293FT cells were cultured in DMEM supplemented with 10% FBS, 1% P/S, and 10% glutamine. Both cell lines were maintained at 37°C in a humidified incubator containing 5% CO_2_. Table S1 summarizes key reagents.

### 2.3 Viruses

SARS‐CoV‐2 variants were obtained from the National Culture Collection for Pathogens, Korea Disease Control and Prevention Agency (KDCA, Table S2).

Viruses were cultured in Vero E6 cells, and once cytopathic effects exceeded 80%, supernatants were harvested and stored at −80°C.

### 2.4 Isolation of Single B Cells

PBMCs from donors with neutralizing sera were used for B‐cell isolation. Cells were washed with Dulbecco's phosphate‐buffered saline (DPBS) containing 1% FBS and 2 U/mL DNase I. The cells were stained using the LIVE/DEAD Fixable Blue Dead Cell Stain Kit and incubated for 15 min with the following antibodies: CD3‐BV421, CD4‐BV421, CD8a‐BV421, CD14‐BV421, CD20‐PerCP cy5.5, and IgG‐FITC [15, 16]. Subsequently, the cells were incubated with antigen probes, S‐type S1‐PE or RBD‐PE, and G‐type S1‐PE for 20 min. Antigen‐specific single memory B cells were sorted into 96‐well PCR plates containing lysis buffer using a FACS Aria II (BD Biosciences, Franklin Lakes, NJ, USA). Data were analyzed using FlowJo software (TreeStar, Oregon, USA). The PCR plates were frozen at −80°C.

### 2.5 Generation of Human Monoclonal Antibodies

The variable regions of the IgG heavy and light chains were separately cloned into pcDNA3.1(+) expression vectors containing an IL‐2 signal sequence and the human IgG1 CH constant region. Transient transfection was performed in 293FT cells using FuGENE transfection reagent (Promega Corporation, Madison, WI, USA) in accordance with the manufacturer's instructions. Heavy chain plasmid, light chain plasmid, and transfection reagent were mixed at a ratio of 1.5:1:2.5 and co‐transfected into the cells. The cells were maintained in DMEM supplemented with 10% FBS at 37°C in a humidified incubator with 5% CO_2_. Culture supernatants were harvested 4 days post‐transfection, clarified by centrifugation, and subsequently filtered through a 0.22 μm membrane. The antibodies were then purified using a Protein G affinity chromatography column.

### 2.6 Evaluation of Antibody Binding Properties

Binding analyses were performed using ELISA and surface plasmon resonance (SPR). Fourteen recombinant SARS‐CoV‐2 spike antigens (S1 and RBD; Sino Biological) were immobilized on nickel‐coated plates and blocked with PBS containing 0.05% Tween 20 and 5% BSA. Serially diluted antibodies (starting at 1 µg/mL) were added, followed by HRP‐conjugated anti‐human IgG and TMB development, and absorbance was measured at 450 nm. For SPR, binding kinetics between antibodies and S1 or RBD variants were assessed on a Biacore 3000 system. Antigens (20 µg/mL in 5 mM acetate buffer, pH 4.0) were immobilized on a C‐Dex100 chip, and serially diluted antibodies (500–7.81 nM) were injected at 30 µL/min. Sensorgrams were analyzed using BlA evaluation software (v.3.1).

### 2.7 Plaque Reduction Neutralizing Test (PRNT)

Neutralization assays were conducted using Vero E6 cells infected with the SARS‐CoV‐2 virus containing variants. Serially diluted antibodies were mixed with 100 PFU of virus and incubated for 1 h at 37°C before infection. After 1 h, cells were overlaid with minimum essential medium (MEM) containing 2% FBS, 1% P/S, 0.5% agarose, and incubated for 2–3 days at 37°C. Plates were fixed with 4% paraformaldehyde solution (PFA) for 1 h, stained with crystal violet, and plaques counted using ImageJ software (NIH ImageJ; NIH, Bethesda, MD, USA). The 50% inhibitory concentration (IC_50_) was calculated using the GraphPad Prism 9 software (GraphPad Software, Boston, MA, USA). Synergistic, additive, and antagonistic interactions between mAbs in virus neutralization were evaluated using the Chou–Talalay method implemented in CompuSyn software (ComboSyn Inc., Paramus, NJ, USA).

### 2.8 Expression and Purification of SARS‐CoV‐2 RBD and Preparation of Fab–RBD Complexes

The SARS‐CoV‐2 spike RBD (residues Thr333‐Arg‐F541, Wuhan‐Hu‐1) was expressed and purified as previously described [17].

For Fab preparation, purified monoclonal IgGs were digested with papain. Digestion reactions proceeded for 180 min at room temperature (20°C~25°C) and 60 min at 37°C for SR‐23 antibody and CS‐42 antibody, respectively. The reactions were quenched by adding 30 mM iodoacetamide, followed by a 2–3× dilution with PBS. The digest was immediately placed on a Superdex 200 Increase 10/300 GL in PBS to separate full‐length antibodies. Fractions containing Fab, Fc, and papain were pooled and incubated with the RBD, followed by a second gel filtration on the same column. The Fab–RBD complex peak was collected and concentrated to 0.05 mg/mL.

### 2.9 Cryo‐EM Grid Preparation and Data Collection

Cryo‐EM grids were prepared using Quantifoil R 1.2/1.3 Cu 200 mesh grids coated with a 2 nm continuous carbon support layer. Before use, grids were glow‐discharged in air for 10 s at 10 mA using a Pelco system. A 2 μL aliquot of protein sample was then applied to each grid, blotted with a blot force setting of 0 for 3 s, and plunge‐frozen in liquid ethane using a Vitrobot Mark IV equilibrated to 4°C and 100% relative humidity. The frozen grids were initially screened using a Glacios microscopy operating at 200 kV. Large data sets were collected on a Titan Krios G4 equipped with a BioQuantum energy filter and a K3 direct electron detector. Table S3 summarizes additional data set‐specific parameters.

### 2.10 Cryo‐EM Data Processing

All image processing was performed using the cryoSPARC v4.2 [18]. Movie stacks were aligned using the Patch Motion Correction algorithm, and contrast transfer function (CTF) parameters were estimated with patch CTF Estimation. An initial particle set was auto‐picked from 2000 randomly selected micrographs using Gaussian blob pickers with 70 and 120 Å thresholds, followed by iterative 2D classification. Particle stacks were further cleaned through additional rounds of 2D classification before the downstream processing.

#### 2.10.1 SR‐23 Fab–RBD Data Set

Three rounds of 3D ab‐initio classification yielded 29 572 curated particles used to train a Topaz model [19]. The trained model was subsequently applied to the whole data set to extract 1 854 641 particle coordinates. After extraction, particles were cleaned by four rounds of 2D classification and two rounds of ab initio 3D classification (*K* = 4), resulting in a high‐quality subset of 115 041 particles that reconstructed a 3.04 Å map. Focused refinement with a soft mask encompassing the Fv and RBD region further improved the interfacial features, achieving a final resolution of 2.83 Å.

#### 2.10.2 CS‐42 Fab–RBD Data Set

Classes derived from blob‐picked particles were used as templates for autopicking across the whole data set. Multiple 2D classifications cleaned the extracted particles and then subjected them to two ab initio classifications (*K* = 4). In total, 47 068 curated particles were used to train a Topaz model, which identified 946 743 particles across the data set. Following extraction and 2D cleaning, three rounds of ab initio classification yielded 37 804 final particles, which reconstructed a 3.31 Å map. As mentioned previously, focused refinement using an Fv‐RBD mask sharpened the epitope‐paratope region to 3.26 Å.

### 2.11 In Silico Model Building and Structural Analysis

Amino acid sequences of SR‐23 and CS‐42 Fv regions were submitted to the AlphaFold3 server to generate a 3D prediction model [20]. The SARS‐CoV‐2 RBD was modeled using the RBD coordinates from the RDB code 7YCO and trimming them to match the expressed construct. Initial rigid‐body placement of each Fv model and the RBD into the corresponding cryo‐EM density map was performed manually, followed by model rebuilding in Coot v 0.9.8.3 [21]. The models were refined against the cryo‐EM maps using PHENIX‐dev‐4694 real‐space refinement with standard secondary‐structure and Ramachandran restraints [22]. Refinement cycles were interleaved with manual adjustments in Coot until no further improvements in map‐model agreement were observed. Molecular graphics and figure panels were generated using UCSF Chimera X [23]. Model refinement statistics are summarized in Table S3.

### 2.12 Animal Experiment

All animal experiments were performed in an Animal Biosafety Level 3 (ABSL‐3) facility following the guidelines approved by the Institutional Animal Care and Use Committee (IACUC, KDCA‐IACUC‐24‐025) of the KDCA. Female K18‐hACE2 mice (6–8 weeks) were obtained from Jackson Laboratory (Bar Harbor, ME, USA) and randomly divided into six groups. The SARS‐CoV‐2 D614G variant was diluted to 100 LD (10^3.4^ pfu), and the BA.2 and XBB.1.5 strains to 1 × 10^5^ PFU. The combined antibodies (SR‐23 and CS‐42) were mixed 1:1. Before infection, mice were anesthetized intraperitoneally with an anesthetic mixture containing 10 mg/kg Rompun (Bayer) and 10 mg/kg Zoletil (Virbac), followed by intranasal inoculation with the virus. Negative control (NC) mice were not infected. Antibodies (SR‐23, CS‐42, and the antibody combination) were administered intraperitoneally 24 h postinfection at doses of 15 mg/kg or 5 mg/kg. Control groups received an equivalent volume of DMEM (NC) or IgG1 (isotype control). On Day 3 postinfection, lungs were collected for viral titration (*n* = 3/group) and histopathology (H&E staining, *n* = 3/group). Lungs were homogenized in DMEM, centrifuged at 12 000 rpm for 20 min at 4°C. For viral titration, Vero E6 cells were infected with serially diluted lung homogenates (10^−1^–10^−5^) for 1 h, incubated in MEM with 2% FBS, 1% P/S, and 0.5% agarose for 2–3 days, fixed with 4% PFA, stained with crystal violet, and plaques counted to determine virus titer.

### 2.13 Statistical Analyses

Statistical analysis was conducted using GraphPad Prism (GraphPad Software, San Diego, CA, USA). An ordinary one‐way ANOVA was applied, followed by Dunnett's multiple comparison test for post hoc analysis. *p* < 0.05 was considered statistically significant.

## 3 Results

### 3.1 Characterization of Monoclonal Antibodies With High Affinity and ACE2‐Blocking Activity Against SARS‐CoV‐2 Variants

Total four clones (SR‐19, SR‐23, SS1‐24, and CS‐42) were selected based on their binding to the S1 and RBD of the SARS‐CoV‐2 S‐type spike protein (Figure S1).

The binding activity of these antibodies was evaluated by ELISA using spike proteins from multiple SARS‐CoV‐2 variants. All four antibodies bound to early variants up to the Gamma variant. SR‐23, SS1‐24, and CS‐42 also recognized the Delta variant, while only CS‐42 bound Omicron subvariants BA.1 and BA.2 when S1 antigens were used (Figure S2A). When RBD antigens were used, SR‐23 also bound to Omicron variants (Figure S2B), suggesting that the corresponding epitope may be conformationally masked in full‐length S1 proteins.

**Figure 1:**
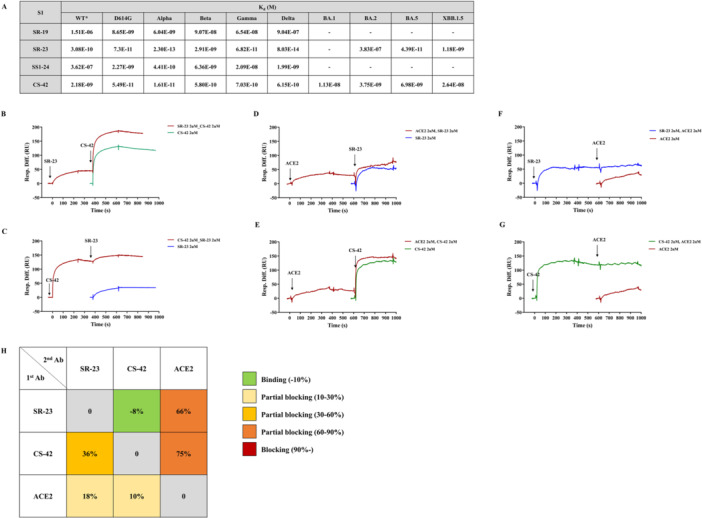
Comprehensive kinetic and epitope characterization of monoclonal antibodies against SARS‐CoV‐2. (A) Binding kinetics between SARS‐CoV‐2 spike protein variants (S1 and RBD) and monoclonal antibodies (SR‐19, SR‐23, SS1‐24, and CS‐42) were analyzed using Bio‐Layer Interferometry (Octet) and Surface Plasmon Resonance (SPR). (B and C) Cross‐competition assays between SR‐23 and CS‐42 were performed through sequential antibody injection using SPR. (D–G) ACE2 competition assays were performed to assess the antibodies' ability to block ACE2 binding. ACE2 was injected first, followed by SR‐23 (D) or CS‐42 (E), or by SR‐23 (F) or CS‐42 (G), which was injected first, followed by ACE2. (H) Summary of binding kinetics and competition results, indicating relative binding strengths and competitive interactions between antibodies and ACE2 across SARS‐CoV‐2 S‐type variants.

SPR analysis was performed to determine the dissociation constants (*K*_d_) between antibodies and S1 or RBD antigens across WT, D614G variant, four VOCs (Alpha, Beta, Gamma, Delta), and four Omicron subvariants (BA.1, BA.2, BA.5, and XBB.1.5). SR‐23 displayed a broad spectrum of binding affinities, ranging from sub‐picomolar interaction with the Delta variant (*K*_d_ = 8.03 × 10^−14^ M) to near‐complete loss of binding to BA.1. A measurable but substantially reduced affinity was observed for BA.2 (*K*_d_ = 3.83 × 10^−7^ M), whereas stronger binding was retained for BA.5 (*K*_d_ = 4.39 × 10^−11^ M) and XBB.1.5 (*K*_d_ = 1.18 × 10^−9^ M), underscoring distinct, lineage‐dependent effects on antibody recognition. CS‐42 exhibited broad binding across all tested variants, including Omicron subvariants, with moderate to high affinity (16.1–54.9 pM for D614G and Alpha; ≥ 11.1 nM for Omicron variants). SR‐19 and SS1‐24 bound to early variants, but not to Omicron subvariants (Figure 1A).

To further assess epitope compatibility, a competitive SPR assay was conducted using recombinant wild‐type SARS‐CoV‐2 spike S1 subunit (Sino Biological) to determine whether SR‐23 and CS‐42 bind competitively or noncompetitively. When SR‐23 was bound first to the S1 subunit and CS‐42 was introduced afterward, an increased binding curve was observed (Figure 1B), indicating that SR‐23 did not interfere with CS‐42 binding. Conversely, when CS‐42 was bound first and SR‐23 was subsequently introduced, only a slight increase occurred (Figure 1C), suggesting that pre‐binding of CS‐42 can partially inhibit SR‐23 binding, possibly through antigen conformational changes or partial epitope disruption.

Subsequently, an ACE2 competition assay showed that pre‐binding of SR‐23 or CS‐42 strongly inhibited ACE2 association (> 66%), whereas pre‐binding of ACE2 did not hinder antibody binding (Figure 1D–H). These findings suggest that both antibodies target epitopes that overlap with or are adjacent to the ACE2‐binding interface.

These findings demonstrate that SR‐23 and CS‐42 exhibit strong, complementary binding across a broad range of SARS‐CoV‐2 variants. CS‐42 showed the widest variant coverage; however, SR‐23 exhibited superior binding affinity for most pre‐Omicron variants. Their partially distinct epitopes and ACE2‐blocking capabilities support their potential use as a broadly neutralizing antibody combination.

### 3.2 Neutralization Efficacy of Single mABs Against SARS‐CoV‐2 Variant Strains

**Figure 2:**
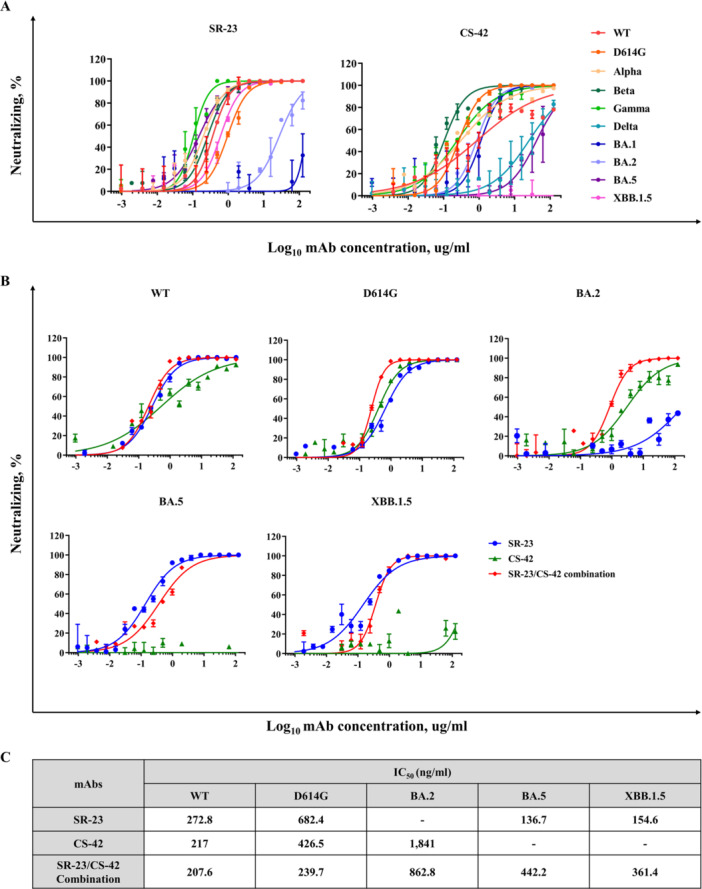
Neutralization assessment of mAbs against SARS‐CoV‐2 variants. (A) Neutralizing activity of individual antibodies, SR‐23 and CS‐42, against SARS‐CoV‐2 variants. (B) Neutralizing activity of the combination of SR‐23 and CS‐42 against SARS‐CoV‐2 variants. (C) IC_50_ values of SR‐23, CS‐42, and their combination, demonstrating enhanced neutralization potency of the combination compared with individual antibodies.

Two mAbs, SR‐23 and CS‐42, which exhibit strong binding affinities to spike antigens of various SARS‐CoV‐2 variants, were selected for neutralization testing. SR‐23 neutralized several variants, including Omicron sublineages; however, it was ineffective against BA.1 and BA.2. Notably, neutralizing activity was restored against BA.5 and XBB.1.5. Among the tested strains, the D614G variant yielded the lowest SR‐23 IC_50_, at 682.4 μg/mL. In contrast, CS‐42 neutralized the WT, D614G, Alpha, Beta, Gamma, BA.1, and BA.2 variants (Figure 2A). Among these, the BA.2 variant exhibited the lowest IC_50_ value, measured at 1841 ng/mL. Comparing these neutralization results with the binding affinity data suggests that neutralization efficacy is not solely determined by binding affinity and that structural or conformational factors may critically influence antibody efficacy.

### 3.3 Enhanced Neutralizing Efficacy Through Antibody Combination

Given that SR‐23 and CS‐42 exhibit distinct neutralizing profiles against Omicron variants, their combined efficacy was evaluated to determine whether they complement each other in neutralizing SARS‐CoV‐2 variants.

Combination analysis demonstrated a synergistic effect of the two antibodies against the WT and D614G variants (Figure S3). For these variants, which are neutralized by both SR‐23 and CS‐42, the IC_50_ values of the combination were 207.6 ng/mL and 239.7 ng/mL, respectively, indicating a modest improvement in neutralizing potency compared with each antibody alone (up to 2.8‐fold under the tested conditions). In contrast, for the BA.2, BA.5, and XBB.1.5 variants—where only one of the two antibodies retained measurable neutralizing activity—the IC_50_ values of the combination were 862.8 ng/mL, 442.2 ng/mL, and 361.4 g/mL, respectively. These results indicate variant‐dependent functional complementarity, whereby neutralization is primarily mediated by the antibody that retains activity against the corresponding variant (Figure 2B,C).

Notably, for BA.2, the enhanced neutralization observed for CS‐42 in the combination may be attributed to a potentiating effect of SR‐23, which, despite lacking intrinsic neutralizing activity against this variant, may contribute through conformational modulation or increased epitope accessibility. For BA.5 and XBB.1.5, SR‐23 retained neutralizing activity, whereas CS‐42 showed minimal or no effect. Nevertheless, the combination of SR‐23 and CS‐42 enabled neutralization across all tested variants, thereby expanding the overall breadth of activity against antigenically diverse strains.

These findings support a model in which the two antibodies compensate for each other's escape vulnerabilities, rather than exhibiting strictly defined pharmacological synergy.

### 3.4 Cryo‐EM Structures of Fabs in Complexes With SARS‐CoV‐2 RBD

**Figure 3:**
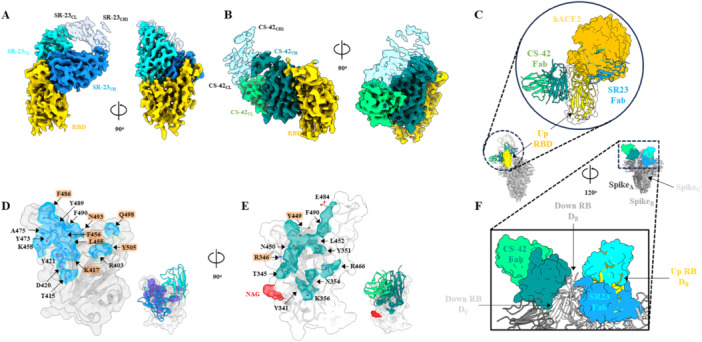
Cryo‐EM analysis of SR‐23 and CS‐42 Fab–RBD complex. (A and B) Cryo‐EM 3D reconstruction of SR‐23 (blue) and CS‐42 (green) Fabs bound to the SARS‐CoV‐2 RBD (yellow), shown from side and top view, illustrating distinct binding orientations. Heavy‐chain variable (VH) and light‐chain variable (VL) domains are labeled, with constant domains (CH1 and CL) labeled in black. (C) Composite model of SR‐23 and CS‐42 Fabs bound simultaneously to the RBD trimer, highlighting nonoverlapping footprints and compatible geometries. The human ACE2 receptor (light yellow) is overlaid for reference. Surface representations of RBD (gray) with mapped epitope residues for SR‐23 (blue, D) and CS‐42 (teal, E), defined as residues with atoms within 4 Å of Fab atoms. Mutation hotspots observed in early Omicron are highlighted in orange. Visible glycans (*N*‐acetylglucosamine, NAG) are displayed in red spheres. (F) Magnified view of the spike trimer model showing simultaneous engagement of SR‐23 and CS‐42 Fabs on adjacent RBDs. SR‐23 binds an up‐positioned RBD, whereas CS‐42 recognizes a neighboring down‐positioned RBD, indicating that the two antibodies can occupy spatially distinct epitopes without major steric overlap.

The cryo‐EM structures of each Fab in complex with the SARS‐CoV‐2 WT RBD were determined to investigate the broad neutralizing activity of these antibodies and elucidate the molecular basis for their differing efficacies. To mitigate the preferred orientation generated during vitrification, holey carbon grids coated with a continuous 2 nm ultrathin carbon film were used. Iterative three‐dimensional classification yielded high‐quality reconstructions of the SR‐23 Fab–RBD and CS‐42 Fab–RBD complexes at 3.2 and 2.8 Å resolution, respectively (Figure S4). The cryo‐EM density maps were of sufficient quality for accurate atomic model building, and both Fabs were well resolved over their complementarity‐determining regions (CDRs) (Figure 3A,B).

SR‐23 and CS‐42 recognized distinct, nonoverlapping epitopes on the RBD (Figure 3C, RBD‐aligned Fab structures). SR‐23 binds to the core of the receptor‐binding motif (RBM), directly occluding the ACE2‐binding ridge, whereas CS‐42 engages a conserved surface on the outer RBD, anchored via multiple salt bridges. This structural segregation explains the complementary neutralization patterns and provides a mechanistic basis for the synergistic activity observed in the combination assays. SR‐23 contacts ACE2‐interface residues, including K417, F456, Y489, Q493, F490, and Y505, through HCDR1‐mediated multiple hydrogen bonds and hydrophobic packing around F456 and Y489, burying approximately 1972 Å of surface area (Figure 3D). This extensive footprint accounts for its sub‐picomolar affinity for early strains and Delta, as well as its sensitivity to Omicron BA.1/2 substitutions at K417, Q493, and Y505. In contrast, CS‐42 buries approximately 2045 Å^2^ across a spatially distinct, invariant epitope that includes R346, K356, Y449, Y451, N450, and R466, with key electrostatic interactions (R346‐HCDR3 and K356‐HCDR1) stabilizing binding (Figure 3E). This footprint avoids mutation hotspots in the initial Omicron lineages, consistent with its retained neutralization breadth against BA.1/2.

The fab‐binding orientation reinforced this complementarity. SR‐23 adopts a near‐vertical approach, characteristic of Class 1 antibodies (Figure 3C), and binds exclusively to the “up” RBD conformation (Figures 3F and S5A), enabling potent receptor blocking but rendering it vulnerable to RBM substitutions in Omicron variants [24]. By contrast, CS‐42 binds laterally, similar to class 3 antibodies, targeting an epitope accessible in both “up” and “down” RBD states, allowing binding with all protomers within the trimer (Figures 3C and S5B).

To further assess the mechanism of CS‐42, its binding orientation was compared with that of the well‐characterized Class 3 antibody 35B5. Structural superimposition of the two Fab variable domains revealed an overall root mean square deviation (RMSD) below 1.0 Å, indicating highly similar binding orientations and paratope configurations (Figure S6A). This close resemblance suggests that CS‐42 neutralizes the spike through a mechanism analogous to that of 35B5. Given previous studies' findings that Class 3 antibodies can modulate spike conformations or stability [25], the impact of CS‐42 binding on the stability‐enhanced HexaPro spike trimer was evaluated [26]. Cryo‐EM micrographs obtained after in vitro incubation with CS‐42 showed a marked increase in particle heterogeneity and a substantial fraction of disassembled or structurally distorted spike molecules, indicating that CS‐42 binding destabilized the pre‐fusion trimer (Figure S6B,C).

### 3.5 Evaluation of Therapeutic Efficacy in Animal Models

**Figure 4:**
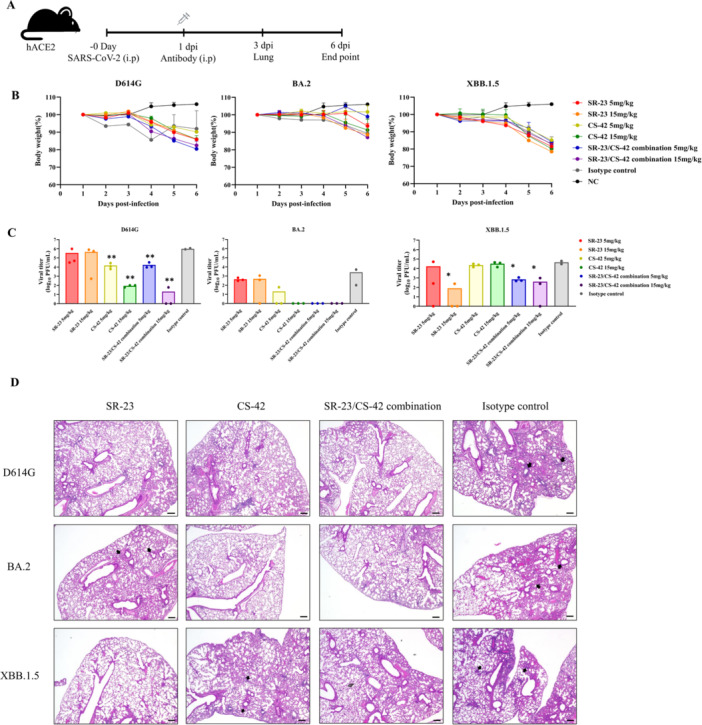
Evaluation of antibody therapeutic efficacy in animal models. (A) Schematic representation of the animal experiment. Mice were infected intranasally on Day 0 and monitored daily for body weight until Day 6. (B) Body weight changes of mice from Day 1 to Day 6 postinfection. Infected animals exhibited progressive weight loss compared to mock‐treated controls. (C) Measurement of viral titers in tissues (lung). Virus titers were quantified by plaque assay and expressed as log10 PFU/mL of homogenate. Infected mice (*n* = 3) showed significantly higher viral titers compared with the isotype control (*p < 0.05, **p < 0.01). (D) Histopathological images of the lung at 3 dpi (magnification ×40; scale bar = 200 µm). Infected mice exhibited inflammatory cell infiltration and alveolar damage compared with the isotype control.

SR‐23 and CS‐42 were administered either individually or in combination to hACE2 transgenic mice infected with SARS‐CoV‐2 variants to evaluate therapeutic efficacy. Antibodies were delivered intraperitoneally 1 day postinfection. Body weight was monitored daily, and lung tissues were harvested for virological analyses (Figure 4A).

Loss in body weight was first observed on Day 3 in the D614G‐infected group and on Day 4 in the BA.2‐ and XBB.1.5‐infected groups. Treatment with CS‐42 resulted in the least overall reduction in body weight; however, the differences among antibody‐treated groups were not substantial (Figure 4B). Moreover, no clear correlation was observed between body weight changes and viral titers. Although antibody treatment reduced viral burden, it did not significantly alter infection‐associated weight loss, suggesting that viral clearance and clinical symptoms may not be fully concordant in this model.

Subsequently, viral titers in lung tissues were quantified following antibody treatment. Mice receiving the isotype control exhibited significantly higher viral titers compared with those treated with SR‐23 or the SR‐23/CS‐42 combination. In the D614G‐infected group, no therapeutic effect of SR‐23 was observed on Day 3, whereas CS‐42 significantly reduced viral load from 1.0 × 10^6^ PFU/mL (control) to 8.6 × 10^1^ PFU/mL, representing a > 10 000‐fold decrease. In the BA.2‐infected group, SR‐23 treatment reduced viral titers by approximately 5‐fold, while CS‐42 achieved a > 2000‐fold reduction compared to the control. In the XBB.1.5‐infected group, the therapeutic efficacy of SR‐23 was observed as early as Day 3 (Figure 4C).

Overall, these findings were consistent with in vitro neutralization data, which showed a dose‐dependent reduction in viral load compared to the IgG isotype control group. SR‐23 demonstrated measurable neutralizing activity against D614G variants in cell‐based assays; however, its efficacy was attenuated in animal models.

Histopathological examination of lung tissues collected on Day 3 postinfection from mice infected with the D614G, BA.2, and XBB.1.5 variants revealed multifocal inflammatory cell infiltrates predominantly localized to peribronchiolar and perivascular regions (Figure 4D). These lesions were more pronounced in the isotype control group, which exhibited dense inflammatory infiltration accompanied by partial disruption of alveolar architecture. In contrast, lung sections from mice treated with SR‐23, CS‐42, or the SR‐23/CS‐42 combination showed reduced inflammatory infiltration and relatively preserved alveolar structures. Representative H&E‐stained lung sections from each group are shown in Figure 4D.

## 4 Discussion

SARS‐CoV‐2 evades the efficacy of existing antibody therapies and vaccines through multiple mutations. Among these, highly transmissible SARS‐CoV‐2 variants with pronounced immune‐evasive properties continue to pose a significant threat, particularly to high‐risk populations [27, 28]. Accordingly, there is an urgent need for variant‐specific therapeutics that can prevent disease progression in these vulnerable groups [29, 30, 31]. Combination antibody therapies such as REGEN‐COV, which comprises two mAbs, have been developed and applied to address these challenges [32, 33].

Neutralizing antibodies were isolated from memory B cells of early‐convalescent SARS‐CoV‐2 patients and evaluated for neutralizing potency against multiple viral variants. Two antibodies, SR‐23 and CS‐42, exhibited distinct neutralization profiles against Delta, BA.1, BA.2, BA.5, and XBB.1.5. Notably, their combination confers therapeutic advantages by maintaining effective neutralization across a broad spectrum of variants. These findings indicate that functional complementarity between antibodies contributes to sustained neutralization breadth and supports the rationale for combination‐based interventions against both current and emerging SARS‐CoV‐2 variants.

Neutralization profiles against variant strains were consistent with structural observations. SR‐23 retained high potency against early strains and Delta but showed high sensitivity to Omicron substitutions. In contrast, CS‐42 exhibited broader activity but showed minimal to no neutralization against BA.5 and XBB1.5. This reduction is attributed to substitutions such as R346T and K356T, which directly disrupt electrostatic interactions (R346‐HCDR3 and K356‐HCDR1) essential for epitope recognition. These complementary vulnerabilities suggest that SR‐23 and CS‐42 compensate for each other's escape liabilities.

The two antibodies target distinct epitopes; however, competitive SPR‐binding measurements revealed an asymmetric binding hierarchy to the full‐length S1 trimers. When SR‐23 was pre‐bound to the spike ectodomain, CS‐42 associated readily, indicating that its lateral epitope remained accessible. However, pre‐binding of CS‐42 strongly impaired subsequent SR‐23 association, suggesting that CS‐42 engagement hinders SR‐23 binding to the spike trimer. Based on its class 3‐like binding orientation, this hierarchy suggests that CS‐42 may destabilize RBDs in the “up” conformation. Additionally, both antibodies effectively inhibited ACE2 engagement regardless of the binding order, consistent with structural findings. These findings indicate that CS‐42 broadens neutralization by indirectly blocking ACE2 access, thereby complementing SR‐23's direct RBM‐targeted activity.

Mechanistically, CS‐42 appears to share the same mode of action as the 35B5 antibody [25]. The 35B5 antibody binds a conserved lateral epitope on the RBD, perturbs the N165/N234 glycan switch, disfavors the RBD‐up conformation, and indirectly blocks ACE2 binding. Superimposition of the CS‐42 Fab–RBD complex with the 35B5‐RBD structure yields a backbone RMSD of 0.8 Å, indicating highly similar epitope recognition. This close structural match supports a glycan switch‐disrupting neutralization mechanism for CS‐42 that limits RBD accessibility.

Combined structural, binding, and neutralization analyses demonstrate that SR‐23 and CS‐42 recognize distinct epitopes with contrasting orientations and binding mechanisms. SR‐23 engages the RBM to block ACE2 directly, whereas CS‐42 targets a conserved lateral surface, potentially stabilizing the closed‐spike protein or disrupting its architecture. This structural complementarity underlies their noncompetitive binding and provides a clear mechanistic basis for synergistic neutralization.

The in vivo efficacy of mAb therapy was evaluated in hACE2‐expressing mice, with antibodies administered individually or in combination. The results demonstrated that monotherapy exhibited only limited or negligible effects, whereas combination therapy substantially reduced viral replication in lung tissues. Differences in viral titer reduction among D614G, BA2, and XBB1.5 may reflect variations in pathogenicity observed in mice for each variant [34, 35, 36].

Notably, the combined antibody treatment enhanced neutralization breadth against diverse SARS‐CoV‐2 variants, suggesting increased versatility in responding to viral mutations [37]. These findings are consistent with previous reports linking reduced viral loads to decreased mortality, supporting the therapeutic potential of mAb combinations against both current and emerging SARS‐CoV‐2 variants.

mAbs isolated from patients infected during the early phase of the pandemic retained neutralizing activity against recent variants, including XBB.1.5. This finding contrasts with most commercially available antibody therapeutics, which show reduced efficacy against Omicron variants. While mAbs isolated from individuals with a history of vaccination or prior infection with Omicron variants have been reported to exhibit broad neutralizing activity against subsequent variants [38], few studies have demonstrated comparable activity in antibodies isolated from patients infected during the initial SARS‐CoV‐2 outbreak [39]. These findings may be attributed to the stronger and more diverse immune response elicited by natural infection compared to vaccination. Furthermore, naturally derived antibodies may have been generated through immune mechanisms optimized for effective viral defense. This study primarily focused on evaluating the therapeutic efficacy of antibodies against viral infections; however, their prophylactic potential was not investigated, which may limit the comprehensive assessment of their antiviral potential.

In conclusion, this study's findings advance antibody‐based therapeutic development by characterizing broadly reactive mAbs. However, further research is needed to expand their breadth to address evolving viral evolution, including bispecific antibody engineering and inhalable delivery methods. Moreover, antibodies isolated from infected individuals during the early phase of the pandemic may represent naturally optimized immune responses, offering insights for designing next‐generation therapeutic antibodies with improved clinical efficacy against emerging SARS‐CoV‐2 variants.

## Author Contributions

Conceptualization: Kyung‐Chang Kim, Joo‐yeon Lee, and Hyun‐Joo Kim. Methodology: Hye‐Min Woo, Hansaem Lee, Jeong‐Sun Yang, Hyun‐Joo Kim, and Hyun‐Soo Cho. Investigation: Da Sol Kim, Uijin Kim, Hye‐Min Woo, Eun‐Seong Jo, Min Jeong Noh, So‐Young Lee, and Dong‐Min Wang. Formal analysis and visualization: Da Sol Kim, Uijin Kim, Eun‐Seong Jo, and Byoung Kwon Park. Writing – original draft: Da Sol Kim and Uijin Kim. Writing – review and editing: Hyun‐Joo Kim and Hyun‐Soo Cho.

## Ethics Statement

Ethical approval for this study was obtained from the Institutional Review Board of Seoul National University Hospital (IRB No. 2002‐105‐110).

## Consent

The convalescent patients agreed to provide biospecimens for further diagnostic and scientific research.

## Conflicts of Interest

The authors declare that the antibodies described in this study are covered by granted patents jointly owned by a government institution. The following authors are listed as inventors on these patents: D.S.K., E.S.J., S.Y.L., H.M.W., HS.L., J.S.Y., J.Y.L., and K.C.K.

## Supporting information

Supporting File 1

## Data Availability

Cryo‐EM data for structures have been deposited in the Protein Data Bank (PDB), with accession codes 9XGX and 9XGW.
