# Supplementary material for: Broad Neutralizing Activity of Monoclonal Antibodies Against the Omicron Variants Isolated From Patients With Early Severe Acute Respiratory Syndrome Coronavirus‐2
Source: J Med Virol. 2026 May 13;98:e70969. doi: 10.1002/jmv.70969 (PMC13170414; doi:10.1002/jmv.70969)
Supplement: Supplementary file 1 — Supporting File 1 [file JMV-98-e70969-s001.docx]

**Supplementary Information**

**Figure S1:** Flow cytometric analysis of memory B cells from peripheral blood mononuclear cells of patients with SARS-CoV-2.

**Figure S2:** Binding of antibodies to SARS-CoV-2 antigens measured by ELISA.

**Figure S3:** Synergistic effects of SR-23 and CS-42 in WT and D614G variants

**Figure S4:** Representative cryo-EM results of the SR-23 and CS42 Fab-RBD complex.

**Figure S5:** Structural alignment of Fab-RBD complexes with the prefusion SARS-CoV-2 spike trimer.

**Figure S6:** Structural comparison of CS-42 and 35B5, and representative cryo-EM micrographs of the HexaPro spike trimer.

**Table S1:** Key reagents.

**Table S2:** List of SARS-CoV-2 variants.

**Table S3:** Cryo-EM data collection and refinement statistics.


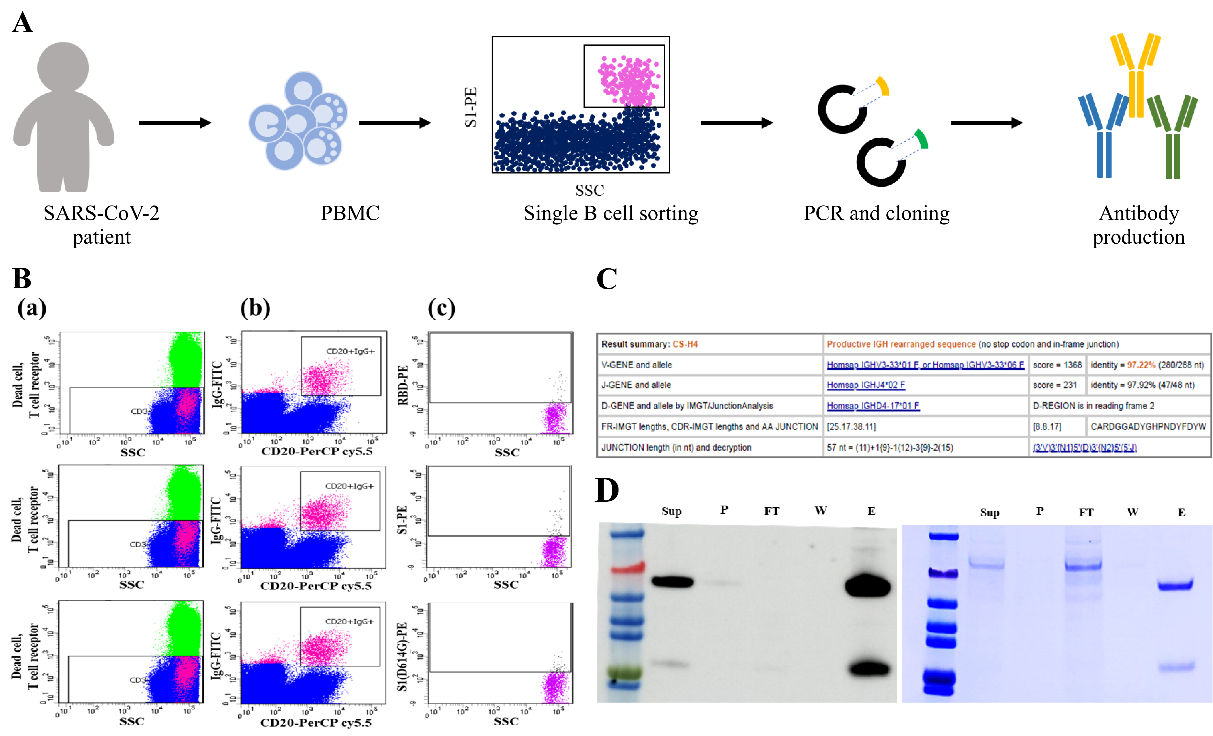


**Figure S1. Flow cytometric analysis of memory B cells from peripheral blood mononuclear cells of patients with SARS-CoV-2. (**A). Schematic of antibody generation: single B cells binding to SARS-CoV-2 antigens were sorted from PBMCs of convalescent patients, followed by gene amplification and generation of candidate antibodies. (B). Single B cell sorting for spike and RBD binding: (a) Negative selection of dead cells and T-cell receptor is shown in P2. (b) Q2 indicates two color staining with anti-IgG/anti-CD20 from negative selected B cells. (c) B cells binding S1 and RBD in the Q2 region were set as Not P3 and then sorted into 96-well plates. In total, 96 events were collected. (C). IMGT analysis of retrieved antibody genes. (D). Expression and purification of candidate antibodies: antibodies were generated in 293FT cells and purified using Protein G; expression and purity were confirmed by Western blot (left) and SDS-PAGE (right).


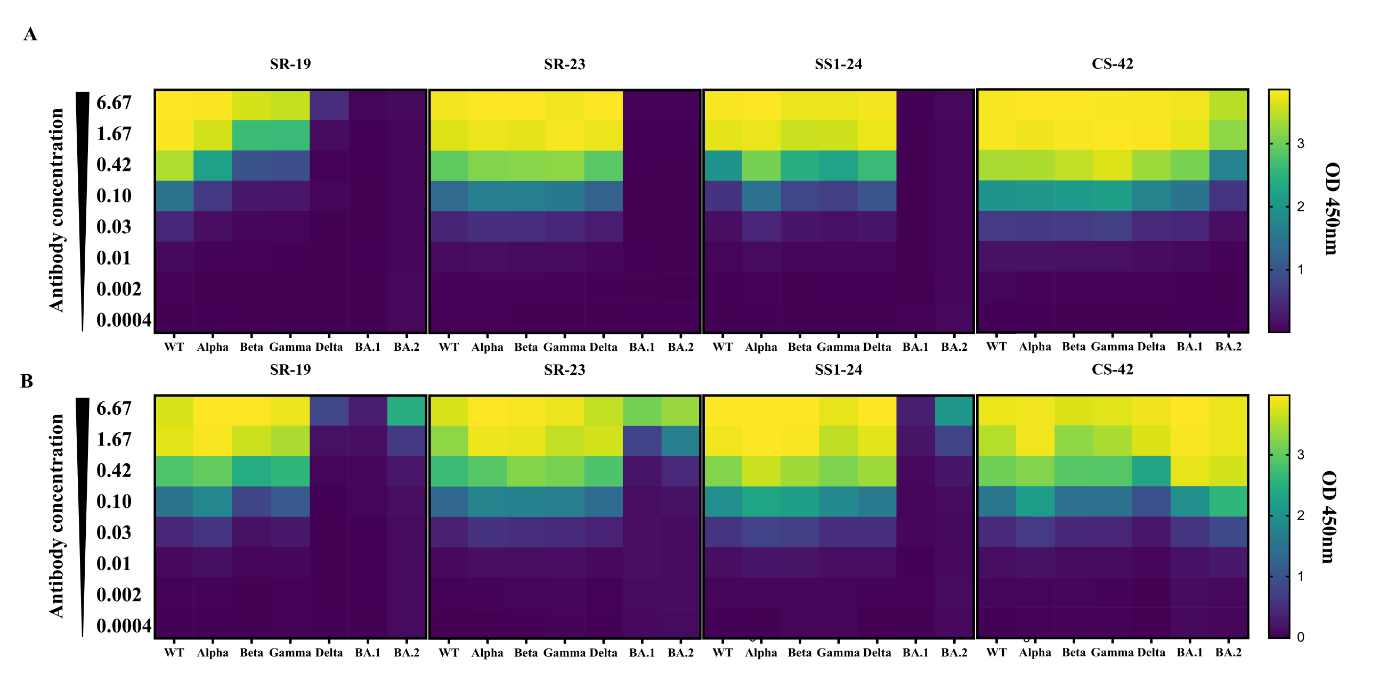


**Figure S2. Binding of antibodies to SARS-CoV-2 antigens measured by ELISA.** The antibodies were tested for binding to the spike protein using (A) the S1 subunit and (B) the RBD as coating antigens. Binding was quantified by measuring the absorbance at 450 nm.


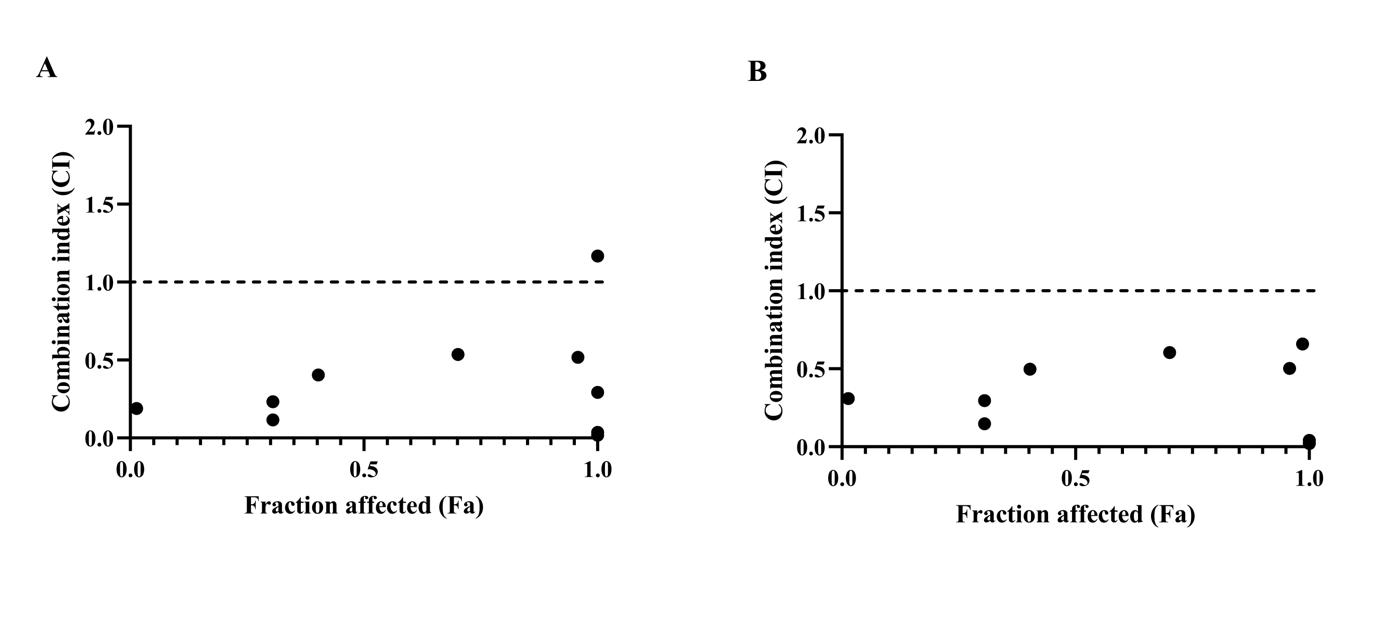


**Figure S3:** Synergistic effects of SR-23 and CS-42 in WT and D614G variants. (A) WT and (B) D614G combination index (CI) plots showing CI values as a function of fraction affected (Fa). CI values were calculated using the Chou–Talalay method implemented in CompuSyn software. A CI of 1 indicates an additive effect, CI < 1 indicates synergism, and CI > 1 indicates antagonism.

**
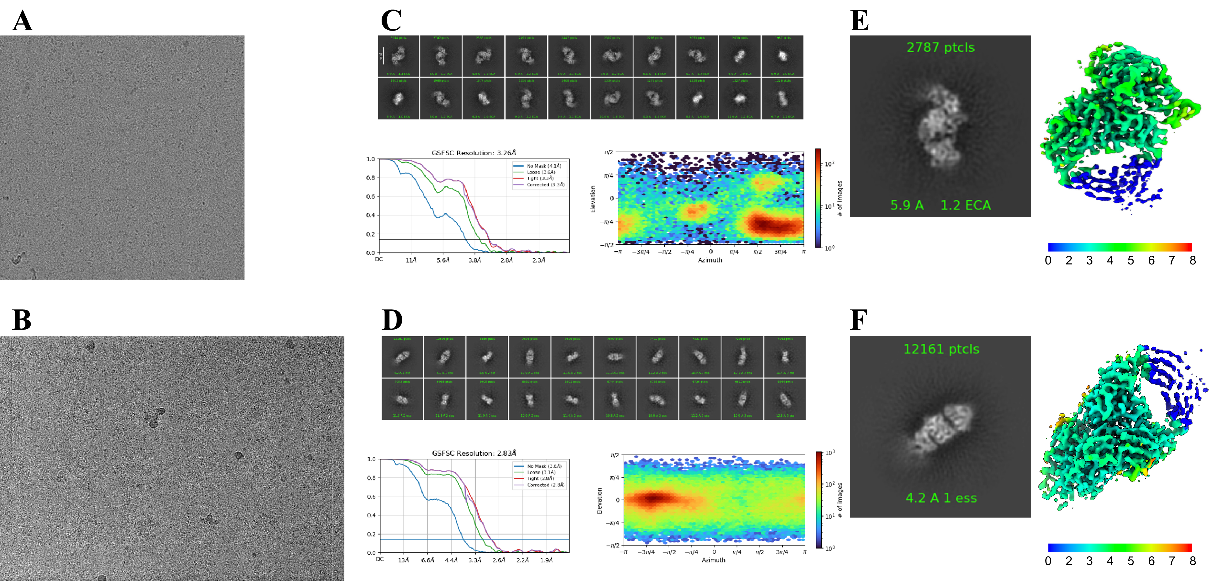
**

**Figure S4. Representative cryo-EM results of the SR-23 and CS42 Fab-RBD complex.** (A–B) Representative cryo-EM micrographs of SR-23 Fab-RBD (A) and CS-42 Fab-RBD *(*B) complexes*.* (C–D) Image processing and validation summaries for SR-23 (C) and CS-42 (D) datasets, showing representative 2D class averages (top), gold-standard Fourier shell correlation (GSFSC) curves with estimated resolution, and viewing direction distribution plots (bottom right). (E–F) Representative 2D classes and final cryo-EM 3D reconstructions of SR-23 Fab-RBD (E, 3.2 Å) and CS-42 Fab-RBD (F, 2.9 Å) complexes, colored by local resolution. Local resolution estimation was performed using a soft mask encompassing the Fv-RBD region.

**
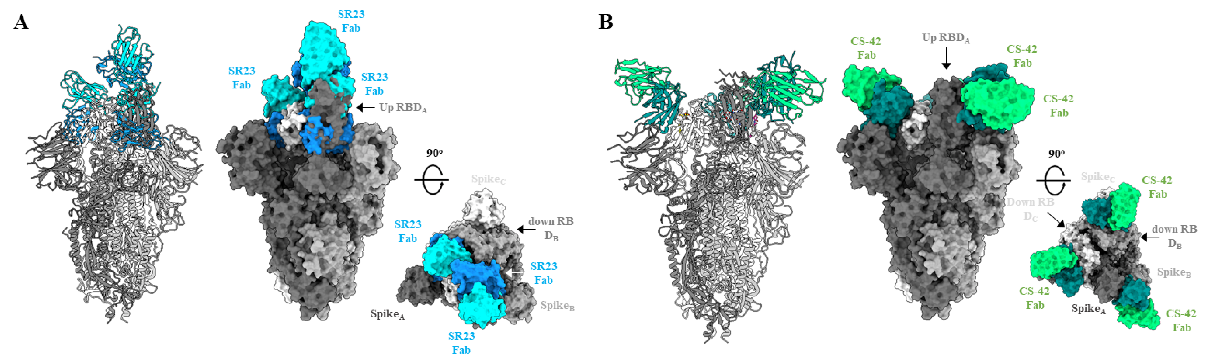
**

**Figure S5. Structural alignment of Fab-RBD complexes with the prefusion SARS-CoV-2 spike trimer.** (A–B) Three copies of the SR-23 (A) and CS-42 (B) Fab-RBD complexes were superimposed onto the 1-up/2-down spike ectodomain trimer. The root-mean square deviation (RMSD) values for the aligned RBDs were 1.051 Å and 1.017 Å for SR-23 and CS-42 complexes, respectively. Spike trimers are shown in gray, with Fabs colored blue (SR-23) or green (CS-42). Left: cartoon representation of the aligned models; middle and right, surface representations rotated by 90^o^. SR-23 binds exclusively to the “up” RBD, occluding the ACE2 binding ridge, whereas alignment to the “down” RBDs results in steric clashes with adjacent protomers. In contrast, CS-42 targets a lateral RBD epitope accessible in both up and down conformations.


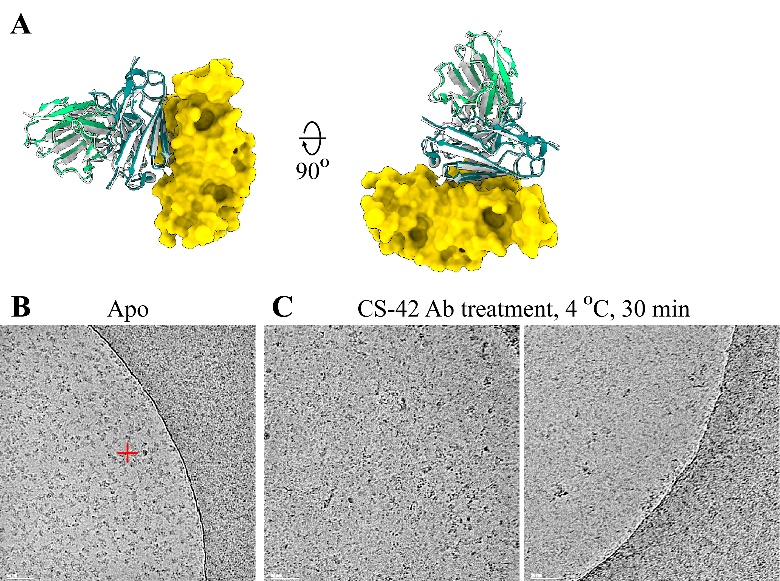


**Figure S6. Structural comparison of CS-42 and 35B5, and representative cryo-EM micrographs of the HexaPro spike trimer.** (A) Structural superimposition of CS-42 and 35B5 (white) Fab variable domains (PDB code: 7WM0) bound to the RBD (yellow surface). The two antibodies adopt highly similar binding orientations and paratope configurations, with an overall RMSD of 0.948 Å. Side and top views are shown. (B–C) Representative cryo-EM micrographs of the HexaPro spike trimer before (B) and after (C) the treatment of CS-42. The HexaPro spike trimer proteins were treated with CS-42 full-length antibody for 30 min at 4^o^C before grid preparation. Scale bar, 50 nm

**Table S1. Key reagents.**

| **Category** | **Name** | **Supplier** | **Catalog No.** |
| --- | --- | --- | --- |
| Cell | VeroE6 | ATCC | CRL-1586 |
| Cell | 293FT | Thermo Fisher | R70007 |
| Media | DMEM | Thermo Fisher | 11995-065 |
| Media | MEM | Thermo Fisher | 11935-045 |
| Reagent | FBS | Thermo Fisher | 10082-147 |
| Reagent | P/S | Thermo Fisher | 15140-122 |
| Reagent | DNaseI | Roche | 10104159001 |
| Buffer | DPBS | Thermo Fisher | 14190144 |
| Antibody | CD3-BV421 | BD biosciences | 562877 |
| Antibody | CD4-BV421 | BioLegend | 317434 |
| Antibody | CD8a-BV421 | BioLegend | 301036 |
| Antibody | CD14-BV421 | BioLegend | 301830 |
| Antibody | CD20-PerCP cy5.5 | BioLegend | 302326 |
| Antibody | IgG-FITC | BD biosciences | 555786 |
| Antibody | Human IgG1 | Bioxcell | BE0297 |
| Antigen | S1-PE | Sinobiological | 40591-V08H |
| Antigen | RBD-PE | Sinobiological | 45092-V08H |
| Antigen | S1-PE (G type) | Cusabio | MP3324GMY |
| Antigen | Wild-type (Wuhan-Hu-1) S1 | Sinobiological | 40591-V08H |
| Antigen | Alpha (B.1.1.7) S1 | Sinobiological | 40591-V08H12 |
| Antigen | Beta (B.1.351) S1 | Sinobiological | 40591-V08H14 |
| Antigen | Delta (B.1.617.2) S1 | Sinobiological | 40591-V08H90 |
| Antigen | Omicron BA.1 S1 | Sinobiological | 40591-V08H85 |
| Antigen | Omicron BA.2 S1 | Sinobiological | 40591-V08H91 |
| Antigen | XBB.1.5 S1 | Sinobiological | 40591-V08H98 |
| Antigen | Wild-type (Wuhan-Hu-1) RBD | Sinobiological | 40592-V08H |
| Antigen | Alpha (B.1.1.7) RBD | Sinobiological | 40592-V08H82 |
| Antigen | Beta (B.1.351) RBD | Sinobiological | 40592-V08H85 |
| Antigen | Delta (B.1.617.2) RBD | Sinobiological | 40592-V08H90 |
| Antigen | Omicron BA.1 RBD | Sinobiological | 40592-V08H122 |
| Antigen | Omicron BA.2 RBD | Sinobiological | 40592-V08H123 |
| Antigen | XBB.1.5 RBD | Sinobiological | 40592-V08H135 |
| Transfection reagent | X-tremeGENE HP | Roche | 063665460 |
| Expression vector | pcDNA3.1 | Genescript | V79020 |
| Protein purification | Protein G column | Cytiva | 17061805 |
| Plate | Nickel-coated plate | Thermo Fisher | 15442 |
| RNA extraction kit | Viral Total Nucleic Acid kit | Promega | AS1330 |
| RT-qPCR kit | PowerChek SARS-CoV-2 | Kogenebiotech | IR6902S |
| FACS viability | LIVE/DEAD Blue | Life Technologies | L23105 |
| Fixative | Paraformaldehyde 4% | bioseasang | BP031 |
| Stain | Crystal violet | Sigma-Aldrich | V5265 |

**Table S2. List of SARS-CoV-2 variants.**

| **Variant name** | **GISAID**  **(accession ID)** | **RBD variant regions** |
| --- | --- | --- |
| Wild type  (A) | BetaCoV/Korea/KCDC03/2020  (EPI_ISL_407193) |  |
| D614G  (B.1.1.119) | hCoV-19/Korea/KCDC9349/2020  (EPI_ISL_812963) | D614G |
| Alpha  (B.1.1.7) | hCoV-19/Korea/KDCA0838/2020  (EPI_ISL_738139) | H69del, V70del, Y144del, N501Y, A570D, D614G, P681H, T716I, S982A, D1118H |
| Beta  (B.1.351) | hCoV-19/Korea/KDCA0463/2020  (EPI_ISL_762992) | L18F, L54F, D80A, D215G, L242del, A243del, L244del, K417N, E484K, N501Y, D614G, A701V |
| Gamma  (P.1) | hCoV-19/Korea/KDCA95637/2021  (EPI_ISL_1622497) | L18F, T20N, P26F, D138Y, T167I, R190S, K417T, E484K, N501Y, D614G, H655Y, T1027I, V1176F |
| Delta  (B.1.617.2) | hCoV-19/Korea/KDCA5439/2021  (EPI_ISL_2887353) | L5F, T19R, G142D, E156G, F157del, R158del, L452R, T478K, Q613H, D614G, P681R, D950N |
| Omicron  BA.1 | hCoV-19/Korea/KDCA18126/2021  (EPI_ISL_6959993) | A67V, H69del, V70del, T95I, G142D, V143del, Y144del, Y145del, N211del, L212I, G339D, S371L, S373P, S375F, K417N, N440K, G446S, S477N, T478K, E484A, Q493R, G496S, Q498R, N501Y, Y505H, T547K, D614G, H655Y, N679K, P681H, N764K, D796Y, N856K, Q954H, N969K, L981F |
| Omicron  BA.2 | hCoV-19/Korea/KDCA61368/2022  (EPI_ISL_13086512) | T19I, L24del, P25del, P26del, A27S, G142D, V213G, G339D, S371F, S373P, S375F, T376A, D405N, R408S, K417N, S477N, T478K, E484A, Q493R, Q498R, N501Y, Y505H, D614G, H655Y, N679K, P681H, N764K, D796Y, Q954H, N969K |
| Omicron  BA.5 | hCoV-19/Korea/KDCA61371/2022  (EPI_ISL_13086516) | T19I, L24del, P25del, P26del, A27S, H69del, V70del, G142D, V213G, G339D, S371F, S373P, S375F, T376A, D405N, R408S, N440K, L452R, S477N, T478K, E484A, F486V, Q498R, N501Y, Y505H, D614G, H655Y, N679K, P681H, N764K, D796Y, Q954H, N969K |
| Omicron XBB.1.5 | hCoV-19/Korea/KDCA0002/2022  (EPI_ISL_16852232) | T19I, L24del, P25del, P26del, A27S, V83A, G142D, Y144del, H146Q, Q183E, V213E, G252V, G339H, R346T, L368I, S371F, S373P, S375F, T376A, D405N, R408S, K417N, N440K, V445P, G446S, N460K, S477N, T478K, E484A, F486P, F490S, Q498R, N501Y, Y505H, D614G, H655Y, N679K, P681H, N764K, D796Y, Q954H, N969K |

**Table S3. Cryo-EM data collection and refinement statistics.**

| **Parameter** | **Datum for the indicated protein** | |
| --- | --- | --- |
|  | **SR-23 (9XGX)** | **CS-42 (9XGW)** |
| EM data collection statistics | | |
| EMDB no. | EMD-66861 | EMD-66860 |
| Microscope | FEI Titan Krios | FEI Titan Krios |
| Voltage (kV) | 300 | 300 |
| Detector | Gatan K3 | Falcon 4i |
| Magnification (nominal) | 105,000 | 130,000 |
| Pixel size (Å/pix) | 0.828 | 0.938 |
| Flux (e−/pix/s) | 8.50 | 7.97 |
| Frames per exposure | 51 | 34 |
| Exposure (e−/Å^2^) | 67.6 | 60 |
| Defocus range (μm) | -1.7, -1.5, -1.3, -1.1, -1.0, -0.8, -0.7 | -2.0, -1.8, -1.6, -1.4, -1.2, -1.0 |
| Micrographs collected | 13,383 | 3,108 |
| Particles extracted/final | 115,041 | 37,804 |
| Symmetry imposed | C1 | C1 |
| Unmasked resolution at 0.5/0.143 FSC (Å) | 3.3/2.8 | 3.6/2.2 |
| Masked resolution at 0.5/0.143 FSC (Å) | 3.3/2.9 | 3.5/3.2 |
| Model refinement and statistics | | |
| PDB | - | - |
| Composition | | |
| Amino acids | 417 | 416 |
| Glycans | NAG (1) | NAG (1) |
| Ligand | 0 | 0 |
| Water | 0 | 0 |
| RMSD bonds (Å) | 0.003 | 0.002 |
| RMSD angles (°) | 0.540 | 0.524 |
| Mean B-factors | | |
| Amino acids | 57.49 | 55.91 |
| Glycans | 118.28 | 80.98 |
| Water | 0 | 0 |
| Ramachandran | | |
| Favored (%) | 95.86 | 94.15 |
| Allowed (%) | 4.14 | 5.85 |
| Outliers (%) | 0.00 | 0.00 |
| Rotamer outliers (%) | 0.29 | 0.86 |
| Clash score | 4.56 | 9.44 |
| C-beta outliers (%) | 0.00 | 0.00 |
| CC (mask) | 0.79 | 0.76 |
| MolProbity score | 1.52 | 1.89 |
